# Incidental Finding of Giant Pericardial Lipoma in a Patient Referred for Rectal Bleeding. Differential Diagnosis and Treatment

**DOI:** 10.1002/ccr3.70502

**Published:** 2025-07-15

**Authors:** Sara Campana, Filomena Ferrentino, Marco Torri, Carlo Rostagno

**Affiliations:** ^1^ Medicina Interna 3 AOU Careggi Firenze Italy; ^2^ Dipartimento Medicina Sperimentale e Clinica Università di Firenze Firenze Italy

**Keywords:** cardiac tumors, diagnosis, heart surgery, MRI, pericardial lipoma

## Abstract

Large circumferential tumors encasing the heart are exceedingly rare, and the differential diagnosis include primary pericardial sarcomas, non‐Hodgkin lymphoma, pericardial primitive neuroectodermal tumor, primary pericardial mesothelioma, and exceptionally mature benign lipoma. Lipomas are rare primary heart tumors. Most are epicardial in origin, although they may arise in the myocardium. They are usually soft masses of mature fat tissue encapsulated by a thin layer of fibrous tissue. Signs and symptoms depend on the tumor's location and size, however in several case they are found incidentally at cardiac imaging. In this 48‐year‐old woman referred to the emergency department for severe intestinal bleeding, echocardiography showed a large circumferential hypoechogenic space, and CT confirmed a large mediastinal fat mass encasing the heart. Multimodal imaging allowed differential diagnosis confirmed by histologic examination performed at surgery.

## Graphical abstract

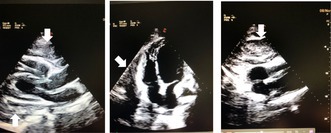

## 1 Introduction

Primary heart tumors are rare conditions, accounting for less than 5% of all cardiac tumors; the remaining 95% of tumors are metastatic localization of solid tumors or hematologic neoplasms. The most common primary cardiac tumors in adults are myxomas 24.4% and lipomas 8.4% [1, 2].

Lipomas of the heart have their origin either from the subendocardium (approximately 50%), pericardium (25%), or from the myocardium (25%). At gross examination, they usually have a homogeneous yellow appearance, are lobulated with smooth borders, and have a thin fibrous capsule. The most prevalent age group affected is approximately 50, with most cases occurring between 40 and 60 [3]. They are frequently located in the left ventricle or right atrium [4]. Signs and symptoms, if present, depend on the size and location of the tumor, as well as the extent of myocardial involvement, and range from chest discomfort to syncope; however, often the discovery is incidental on cardiac imaging. Echocardiography allows the identification of a cardiac mass, its location, mobility, and hemodynamic impact; however, the capability for tissue characterization is limited [5]. Radiologic imaging enables tissue definition. At magnetic resonance (MRI) lipoma has the same signal intensity as subcutaneous fat. Hyperintensity in T1W1 and T2W1, loss of signal with fat suppression, and no significant enhancement post intravenous gadolinium are hallmarks of adipose tissue.

CT scan shows lipoma as a fatty attenuating tumor with a < 0 HU density. They do not show enhancement after contrast administration [6, 7]. CT imaging is useful in the differential diagnosis with liposarcoma, which, in contrast to the lipoma, has a higher HU number than normal subcutaneous fat. Although multimodal imaging suggested a “benign” condition, for large pericardial masses encasing the heart, surgery is recommended because the histopathological examination of excised tissue is essential to confirm the diagnosis and rule out malignancy. Pericardial biopsy alone, in particular for the transcutaneous approach, is associated with a high procedural risk and with a non‐negligible percentage of false negative results. Moreover, growing benign pericardial lipomas can infiltrate the myocardium, and excision at that point may be extremely difficult. We describe a patient with no cardiovascular symptoms, in whom the presence of a large cardiac mass encasing the heart was incidentally identified by imaging studies.

## 2 Case History‐Examination

**FIGURE 1:**
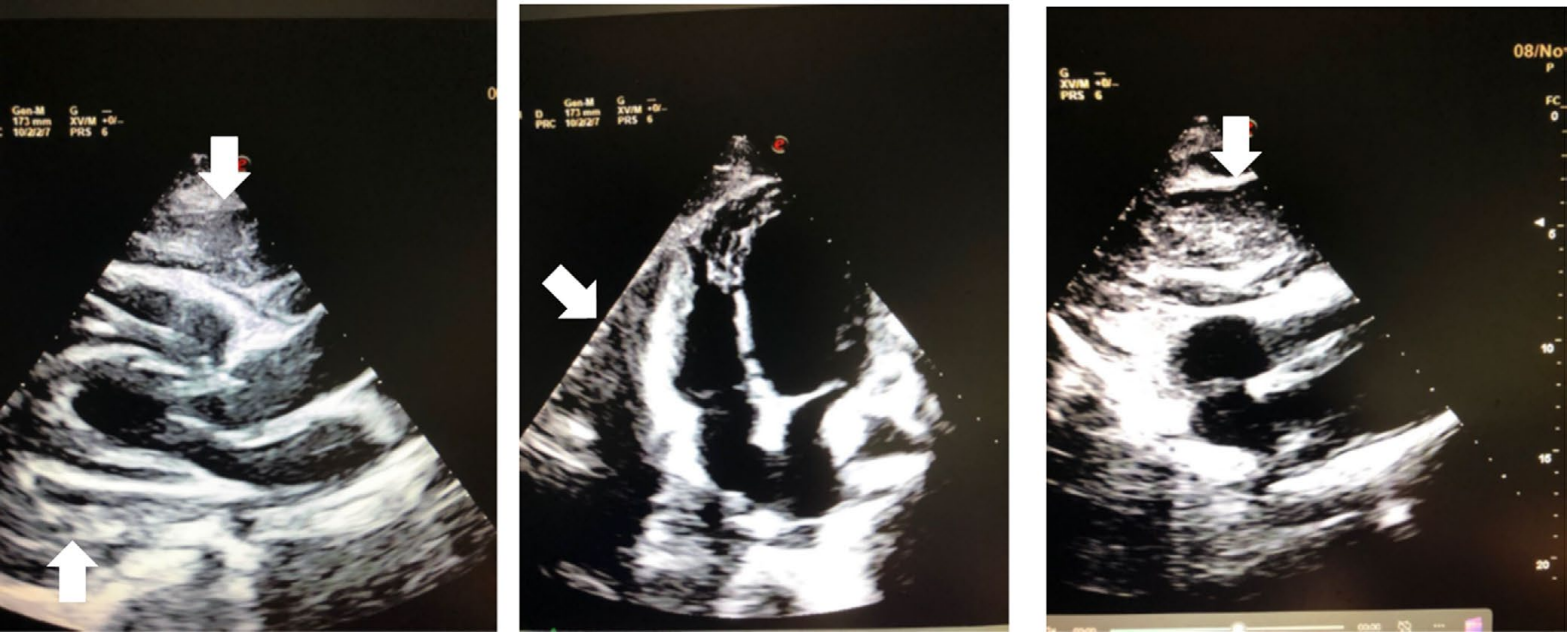
Admission TTE—left: Parasternal long axis view‐center‐apical 4 chamber view—right subxifoid view‐the heart is surrounded by not echo graphic homogenous space of maximal thickness 6 cm.

A 48‐year‐old woman accessed the E.D. for repeated intestinal bleeding. At the hospital admission, blood pressure was 100/50 mmHg, heart rate 65 beats/min, Hb 10 g/dL. She had no cardiac symptoms. Physical examination did not disclose heart murmurs, gallop, or pericardial rubs. The lungs were clear to auscultation. An electrocardiogram showed normal sinus rhythm without remarkable abnormality. She had previous episodes of rectal bleeding for which she underwent colonoscopy and removal of intestinal polyps (histological examination showed tubulo‐villous intestinal adenoma with expressions of high‐grade epithelial dysplasia) for which she has been in endoscopic follow‐up. Echocardiography showed a large circumferential hypoecogenic pericardial space with a maximal thickness of 6 cm (Figure 1).

## 3 Differential Diagnosis, Investigations and Treatment

**FIGURE 2:**
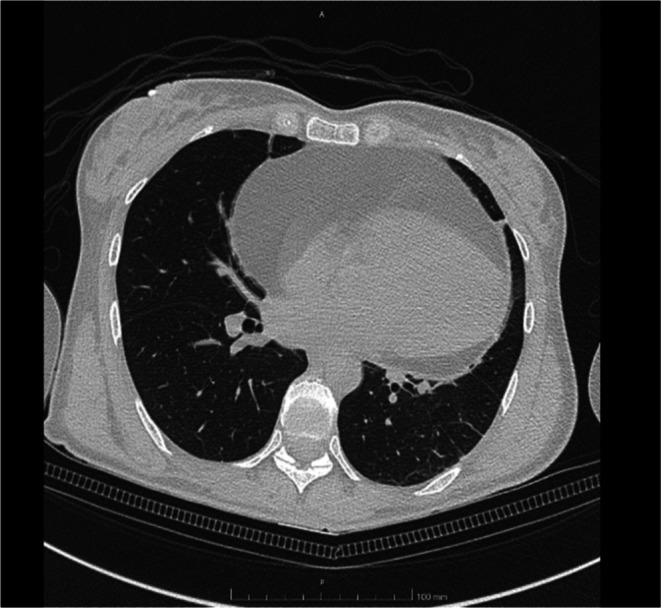
Chest‐CT—This transversa chest CT image shows a large mass (maximum diameters on the axial plane 17 × 11 cm) encasing the heart with mature fat aspect (low density–HU–of the tissue surrounding the heart).

The patient did not show signs or symptoms due to restrictive effects on heart chambers; ECG showed sinus rhythm at 66 b/min. Echocardiographic examination showed a wide pericardial space (maximal thickness 6 cm) surrounding the heart with not homogenous characteristics. Echocardiographic characteristics suggested the suspicion of a large pericardial lipoma; however, it should be differentiated from other space‐occupying pericardial diseases like mesothelioma, pericardial cysts (which appears as an anechoic region) and secondary tumors (often secondary to lymphoma, melanoma, lung cancer). Chest CT showed a huge cardiac mass with an attenuation value identical to that of subcutaneous adipose tissue, which completely surrounded the heart up to the origin of the epiaortic vessels (maximum diameters on the axial plane 17 × 11 cm) (Figure 2). The mass showed some septa with supradense density without contrastographic impregnation. Although MRI could provide several pieces of information in the differential diagnosis with liposarcoma, such as the presence of prominent areas of enhancement associated with non‐adipose lesions, thick or nodular septa, and prominent foci of high T2 signal the margins are often nodular and have a lower fat content (< 75%). Despite its clinical relevance, the patient, however, refused to perform MRI due to claustrophobia.

**FIGURE 3:**
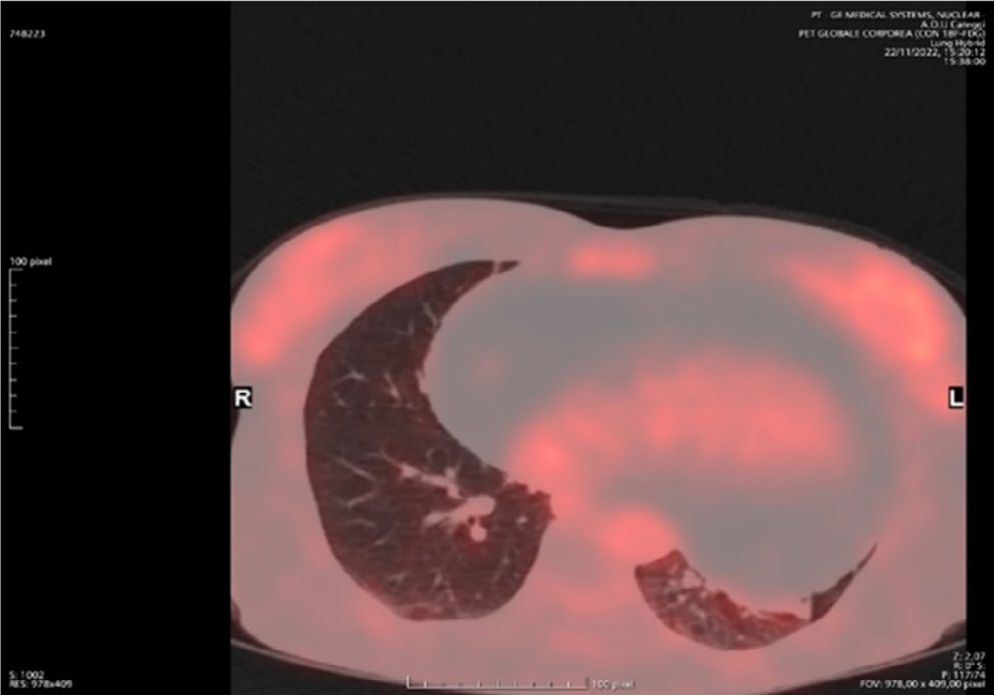
PET scan show no significant metabolic activity in correspondence of the large mediastinal mass surrounding the heart, suggesting the absence of malignant tissue.

Although CT showed findings consistent with a benign lipomatous formation, further information could be obtained by positron emission tomography (PET). PET has 100% sensitivity and 92% specificity for differentiating benign and malignant cardiac tumors. Results from the PET examination of the mass showed minimal/absent mediastinal metabolic activity consistent with the absence of tissue with a high proliferative rate and with the diagnosis of lipoma (SUV max 1.4, hepatic SUV 2.5) (Figure 3).

## 4 Outcome and Follow‐Up

The mass was surgically removed through median sternotomy. At gross examination, it had a lipomatous appearance with a vascularized peduncle on the anterior portion of the epicardium. Surgical resection was complicated by pericardial effusion with only mild hemodynamic impact treated conservatively with steroids. Histological examination confirmed definitively the diagnosis of mature intrapericardial lipoma. Indication for CT periodic follow‐up was given after hospital discharge. Colonoscopy revealed a new intestinal polyp that was removed before heart surgery.

## 5 Conclusion

Cardiac lipomas are rare tumors and they are usually asymptomatic; however, rarely a large circumferential mass encasing the heart is found, and in these patients, an alternative diagnosis should be suspected. Although echocardiography may raise clinical suspicion, differential diagnosis may not be solved by ultrasound examination. Multimodal diagnostic imaging is needed to better define the mass. Chest CT and cardiac MRI offer better characterization of mature fat tissue; moreover, the absence of contrast enhancement allows in almost all cases a differential diagnosis with pericardial neoformations such as liposarcoma, mesothelioma, lymphoma, or secondary neoplasms. Finally, the finding of low metabolic activity at PET examination further suggests the presence of a benign condition. Nevertheless, histologic examination after surgical resection gave a definite diagnostic confirm. The slow growth of the mass may explain the absence of symptoms in our patient; however, heart compression in time might lead to a constrictive pattern. The main surgical difficulty was the radical resection of the mass, associated with a non‐negligible risk of bleeding.

## Author Contributions

**Sara Campana:** writing – original draft, writing – review and editing. **Filomena Ferrentino:** writing – original draft, writing – review and editing, writing – review and editing. **Marco Torri:** supervision, writing – review and editing. **Carlo Rostagno:** conceptualization, data curation, validation.

## Consent

Written informed consent was obtained from the patient to publish this report in accordance with the journal's consent policy.

## Conflicts of Interest

The authors declare no conflicts of interest.

## Data Availability

Data sharing not applicable to this article as no datasets were generated or analyzed during the current study.
